# Community governance and nutrition-oriented cancer care for women: policy pathways to reduce survival disparities

**DOI:** 10.3389/fpubh.2026.1912567

**Published:** 2026-08-17

**Authors:** Liqian Hao, Xiaomin Ma, Yiyan Feng

**Affiliations:** 1 Shanxi Normal University, Taiyuan, China; 2 Shanxi Engineering Vocational College, Taiyuan, China

**Keywords:** cancer nutrition, community governance, food insecurity, health equity, nutrition policy, service integration, survivorship care, women's cancers

## Abstract

Nutrition is increasingly recognized as a modifiable determinant of treatment tolerance, metabolic health, quality of life, and survivorship outcomes among women with cancer. However, nutrition-oriented cancer care is often delivered as an individual-level clinical recommendation, while many determinants of dietary behavior and nutritional vulnerability arise outside oncology clinics. Food insecurity, unequal food environments, limited dietitian availability, fragmented referral systems, low nutrition literacy, transport barriers, and gendered caregiving roles can prevent women with breast, cervical, ovarian, endometrial, and other cancers from receiving timely and effective nutrition support. Community governance provides a policy framework for linking oncology services with primary care, public health agencies, community organizations, food assistance programs, digital platforms, and patient groups. This Mini Review examines how community governance can strengthen nutrition-oriented cancer care for women and reduce survival disparities. We summarize pathways through which community-level conditions influence nutritional status and survivorship, distinguish established evidence from promising implementation models and expert-policy rationale, and propose governance mechanisms for nutrition screening, referral, food support, and follow-up. Community-governed nutrition care should move beyond service volume and evaluate whether nutritionally vulnerable women receive accessible, culturally appropriate, and outcome-oriented support across the cancer continuum.

## 1 Introduction

Women with cancer frequently face nutritional challenges across diagnosis, treatment, rehabilitation, and long-term survivorship. Malnutrition, unintentional weight loss, sarcopenia, obesity, metabolic dysfunction, micronutrient insufficiency, and poor diet quality can influence treatment tolerance, complication risk, physical function, quality of life, and survival-related outcomes (1–6). In breast, endometrial, ovarian, cervical, and other women's cancers, nutritional status also intersects with endocrine state, systemic inflammation, insulin–IGF signaling, adipokine biology, gut microbial metabolism, and body-composition change (2, 7–10). Thus, nutrition is not merely supportive care; it is a biologically plausible and clinically actionable component of cancer survivorship.

Despite this importance, nutrition-oriented cancer care remains inconsistently implemented. Many patients do not receive routine nutrition risk screening, dietitian referral, symptom-responsive dietary counseling, or follow-up monitoring (5, 6, 11). Even when general dietary recommendations are provided, women may be unable to follow them because of food insecurity, financial toxicity, transport barriers, low nutrition literacy, treatment-related symptoms, household caregiving responsibilities, and limited access to healthy foods (12–15). These barriers are particularly relevant for women in low-resource settings, rural areas, underserved urban neighborhoods, and communities with fragmented cancer care systems.

Community governance offers a way to address these limitations. In this context, community governance refers to coordinated decision-making, accountability, and service delivery across oncology providers, primary care, public health agencies, social care organizations, local food systems, community health workers, patient groups, and local governments. It differs from conventional multidisciplinary or integrated care models by extending responsibility beyond the hospital team to include food access, social support, local implementation capacity, and equity monitoring. Rather than treating nutrition as a clinic-only recommendation, community governance frames nutritional care as a cross-sector service pathway that requires screening, referral, food access, education, social support, and outcome monitoring. This Mini Review discusses how community governance can support nutrition-oriented cancer care for women and proposes policy pathways to reduce survival disparities.

### 1.1 Literature search and conceptual scope

We conducted a targeted narrative search of PubMed, Web of Science, Google Scholar, WHO and National Cancer Institute resources, and guideline documents published mainly between 2015 and 2025, while retaining older foundational frameworks where they defined malnutrition, access, integrated care, or community health worker models. Search terms included cancer nutrition, women with cancer, survivorship, malnutrition screening, food insecurity, community health workers, social care, integrated care, food is medicine, nutrition policy, and health equity. We prioritized guidelines, systematic reviews, randomized or pragmatic implementation studies, cohort studies, and policy frameworks relevant to women with cancer or oncology survivorship. Because this is a Mini Review rather than a systematic review, evidence was synthesized narratively and grouped as established evidence, promising implementation evidence, or expert-policy rationale.

Conceptually, community governance overlaps with integrated care, the Chronic Care Model, social determinants of health frameworks, and the WHO integrated people-centered care agenda, but it adds an operational emphasis on local accountability and cross-sector resource coordination (16–20). In this review, established evidence refers to consistent clinical or epidemiological findings supporting nutrition screening, malnutrition management, or food insecurity assessment; promising implementation evidence refers to programs such as community health worker support, navigation, and food assistance that show benefit but require contextual adaptation; expert-policy rationale refers to governance, financing, and equity-monitoring recommendations that remain insufficiently tested in oncology-specific trials.

## 2 Why nutrition-oriented cancer care requires community governance?

Nutrition care in oncology traditionally focuses on clinical assessment and individualized dietary counseling. This remains essential, but it is insufficient when the barriers to adequate nutrition are structural and community-based. A woman receiving chemotherapy for breast or ovarian cancer may be advised to increase protein intake, preserve muscle mass, manage nausea, and maintain body weight. Yet the feasibility of these recommendations depends on income, food availability, household support, transportation, cultural food practices, symptom burden, and access to professional nutrition guidance.

Cancer treatment can reduce dietary intake through anorexia, nausea, dysgeusia, mucositis, dysphagia, diarrhea, constipation, pain, fatigue, depression, and endocrine therapy-related weight change (5, 6, 21). At the same time, women may experience work interruption, increased out-of-pocket medical costs, and reduced household resources. These conditions can shift food choices toward cheaper, energy-dense, nutrient-poor foods or reduce total intake. In hormone-related cancers, excess adiposity and metabolic dysfunction may contribute to inflammatory and endocrine pathways relevant to recurrence risk and comorbidity burden (7–10). Conversely, undernutrition and muscle loss can worsen treatment tolerance and physical function.

These nutritional problems are not distributed evenly. Women living in disadvantaged neighborhoods may face limited availability of fresh foods, unsafe environments for physical activity, inadequate public transportation, and weak primary-care follow-up. Older women, single mothers, migrants, ethnic minorities, and rural residents may have lower access to oncology dietitians or supportive care services. A purely clinic-based model cannot adequately address these social and spatial determinants.

**Figure 1:**
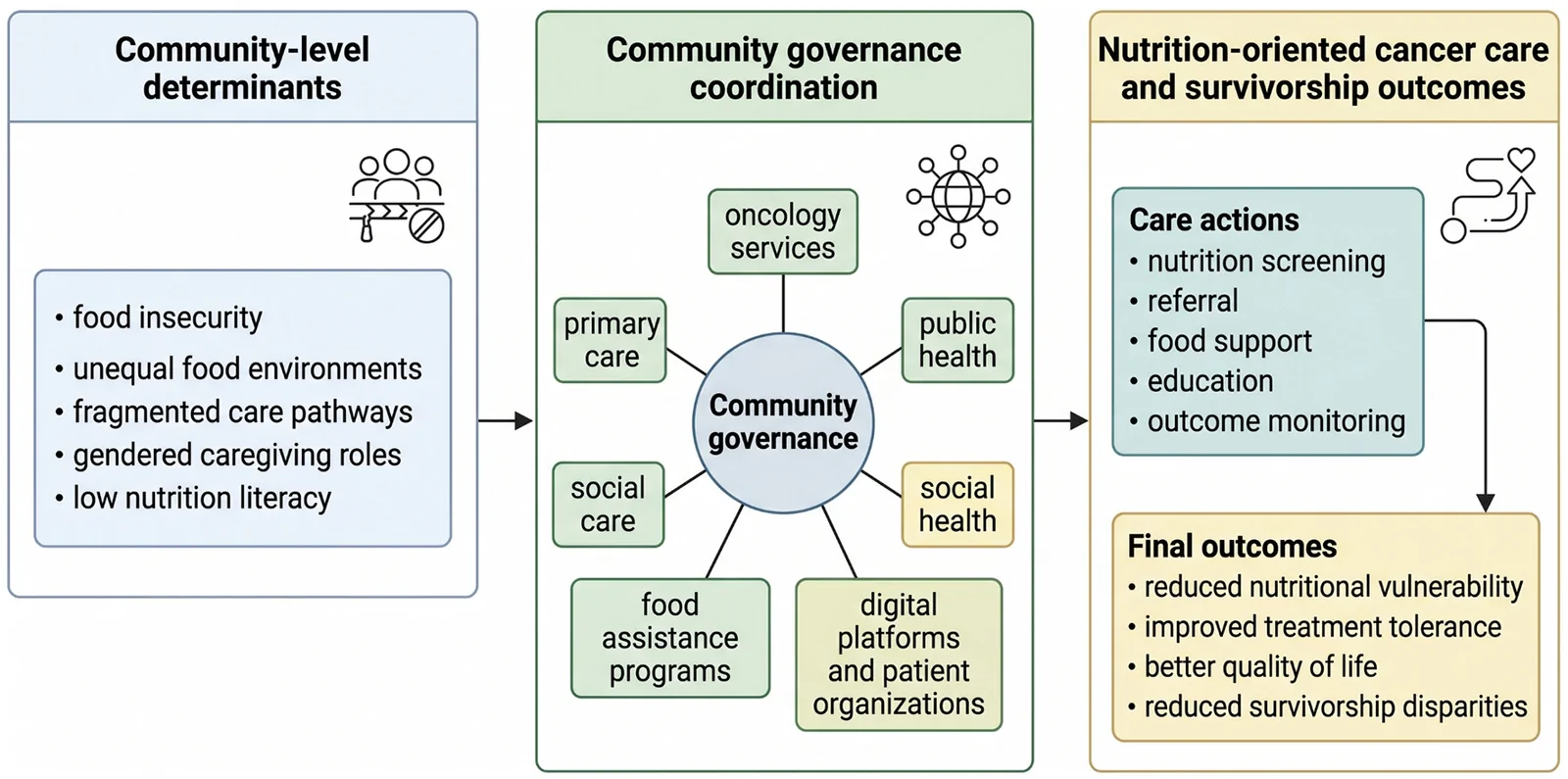
Community governance framework for nutrition-oriented cancer care among women. Community determinants shape nutritional vulnerability and survivorship disparities, while coordinated governance links oncology, primary care, public health, social services, food assistance, digital platforms, and patient organizations to support screening, referral, education, food access, and outcome monitoring.

Community governance is therefore necessary because it expands the unit of intervention. The target is not only the individual patient, but also the service network around her: the oncology clinic that screens for nutritional risk, the community health worker who follows up at home, the primary-care team that monitors comorbidities, the local food program that improves dietary access, and the digital platform that supports symptom-responsive guidance. When coordinated, these actors can transform nutrition care from an optional recommendation into a structured survivorship service. The proposed community governance framework is summarized in Figure 1, and the evidence categories supporting this framework are summarized in Table 1.

**Table 1:** Evidence categories for community-governed nutrition-oriented cancer care for women.

Evidence domain	Representative evidence or framework	Main implication	Current limitation
Nutrition screening and malnutrition care	ESPEN, ACS, GLIM and oncology nutrition guidance support screening, assessment and individualized nutrition intervention (2, 5–7, 21, 28–30).	Established clinical foundation for identifying nutritional vulnerability across treatment and survivorship.	Implementation is inconsistent, especially in outpatient, rural and low-resource settings.
Food insecurity and social needs	Food insecurity is associated with poor diet quality, forgone care and adverse outcomes among cancer survivors (12–15).	Food access should be assessed together with malnutrition risk and treatment symptoms.	Most studies are observational; oncology-specific intervention trials remain limited.
Community health workers and navigation	Community health worker and navigation models have improved care coordination and acute-care use in selected cancer or chronic-care settings (22–26).	Lay and community-based support can extend nutrition follow-up beyond tertiary centers.	Training, supervision and role boundaries must be standardized to avoid unsafe advice.
Food assistance and food-is-medicine programs	Food assistance, vouchers, medically tailored food and community partnerships have plausible value for cancer nutrition support (12, 17, 27, 33).	Nutrition recommendations become actionable when patients can obtain appropriate foods.	Sustainable financing, eligibility criteria and cancer-specific outcomes are often underdeveloped.
Digital and hybrid support	Digital tools can support education and monitoring but may exclude patients with low digital access or literacy.	Hybrid models can combine tele-nutrition, telephone follow-up, community health workers and printed materials.	Digital inequality and privacy concerns can reproduce disparities if governance is weak.
Governance and equity monitoring	Integrated care, social care, WHO people-centered care and access frameworks support cross-sector coordination (16–20, 31, 32).	Nutrition quality should be judged by high-risk patient reach, referral completion and equity-sensitive outcomes.	Few oncology studies test governance models as accountable systems with pre-defined implementation metrics.

## 3 Community-level pathways linking nutrition to survivorship disparities

### 3.1 Food insecurity and unequal food environments

Food insecurity is one of the clearest community-level drivers of nutritional vulnerability. It can reduce access to nutrient-dense foods, delay care-seeking, force trade-offs between food and medications, and increase psychological stress (12–15). For women with cancer, food insecurity may be intensified by treatment costs, job loss, caregiving responsibilities, and the need for specific foods during periods of nausea, weight loss, or immunosuppression. Even in settings where total caloric intake is sufficient, low diet quality can coexist with obesity, diabetes, and micronutrient inadequacy.

Evidence in this domain is strongest for association rather than causality. Food insecurity and financial strain are consistently linked to poorer diet quality and care disruption, whereas evidence that food assistance improves cancer-specific survival remains limited. Randomized and pragmatic trials more often report treatment completion, patient-reported outcomes, food security, or quality-of-life endpoints than long-term survival. This distinction is important because community governance should be evaluated by realistic intermediate outcomes before claims are made about mortality reduction.

Food environments also matter. Communities with limited fresh food availability, high prices for fruits, vegetables, lean proteins, and whole grains, and abundant ultra-processed food outlets may make survivorship-oriented dietary recommendations difficult to implement. This is especially relevant for breast and endometrial cancer survivors, for whom weight management and metabolic health are important, and for patients undergoing active therapy, for whom adequate protein and energy intake are necessary to preserve function.

### 3.2 Fragmented care pathways

Nutrition services often fall between oncology, primary care, and social care. Oncologists may recognize weight loss or symptom-related eating difficulties but lack time for detailed nutrition counseling. Dietitians may be concentrated in tertiary centers, leaving community hospitals and rural clinics underserved. Primary-care providers may manage diabetes, hypertension, or obesity but may not receive timely cancer treatment information. Social workers may address financial stress but may not be integrated with nutrition referral pathways.

This fragmentation creates missed opportunities. A patient with early weight loss may not be referred until severe malnutrition develops. A woman with obesity and endocrine therapy-related weight gain may receive generic advice without structured support. A survivor with food insecurity may be told to improve diet quality without being linked to food assistance. Community governance aims to close these gaps by clarifying roles, standardizing referral criteria, and creating accountable pathways across sectors.

International experience suggests that no single community model is universally transferable. Rural oncology nutrition referral studies, community health worker and patient-navigation programs, food-is-medicine initiatives, and food security governance models in Latin America each address different barriers, including distance, workforce shortages, food affordability, and local accountability (15, 17, 22–27). The implication is that community governance should be adapted to local service capacity rather than copied as a fixed package.

### 3.3 Gendered social roles and caregiving burden

Women's nutritional vulnerability during cancer survivorship is shaped by gendered social roles. Many women continue to manage household food preparation, childcare, eldercare, and family nutrition while undergoing treatment. These responsibilities may reduce time for self-care, medical appointments, exercise, and symptom-responsive eating. In some households, cultural expectations may prioritize feeding family members over meeting the patient's own nutritional needs. Social stigma and misconceptions about “anti-cancer diets” can further complicate eating behavior.

Community governance can address these factors by involving family caregivers, community organizations, women's groups, and patient navigators. Nutrition education should not only provide dietary rules; it should help women negotiate household food practices, manage symptoms, avoid harmful restrictive diets, and access practical support.

## 4 Governance mechanisms for nutrition-oriented cancer care

### 4.1 Routine community-linked nutrition screening

The first policy pathway is routine nutrition screening across the cancer continuum. Screening should occur at diagnosis, before treatment, during chemotherapy or radiotherapy, after surgery, during endocrine therapy, and in survivorship follow-up. Tools such as PG-SGA, NRS-2002, GLIM-based assessment, weight-change monitoring, food insecurity screening, and body-composition assessment can identify risk at different levels (5, 6, 28–30).

Community governance adds two requirements. First, screening should trigger action. A positive screen must lead to dietitian referral, symptom management, food assistance, or social care navigation. Second, screening should be documented and monitored across care settings. Nutrition risk identified in a tertiary hospital should be visible to primary care and community follow-up teams. Without such linkage, screening becomes a documentation exercise rather than a pathway to care.

### 4.2 Risk-stratified referral and service allocation

Not all women require the same level of nutrition support. Community-governed systems can adopt risk-stratified allocation. Low-risk survivors may benefit from brief education on healthy dietary patterns, physical activity, and weight maintenance. Moderate-risk patients may require structured group education, symptom-specific dietary guidance, and follow-up calls. High-risk patients—such as those with severe weight loss, sarcopenia, poor intake, food insecurity, advanced disease, or complex comorbidities—should receive dietitian-led intervention, social support, and closer monitoring.

This approach improves efficiency and equity. Service allocation should be evaluated by whether high-risk women receive timely and effective support, not by the number of educational brochures distributed or dietitian visits recorded. Equity-oriented metrics should include screening coverage, time to referral, food assistance linkage, change in nutrition status, symptom burden, treatment interruption, quality of life, and patient-reported access.

### 4.3 Community health workers and patient navigation

Community health workers, patient navigators, and trained lay supporters can bridge the gap between oncology clinics and households. Their roles may include identifying food insecurity, reinforcing nutrition education, supporting appointment adherence, coordinating transportation, connecting patients with food programs, monitoring symptoms, and communicating concerns to clinical teams (16, 17, 22, 31, 32). These roles are particularly useful in rural or underserved communities where specialist dietitians are scarce.

However, community health workers should not replace dietitians. Their function is to extend the reach of the care team, identify barriers early, and support practical implementation. Training should cover basic cancer nutrition principles, red-flag symptoms, culturally appropriate dietary counseling, food safety, referral criteria, and psychosocial communication. Clear supervision and escalation pathways are needed to avoid unsafe or inconsistent advice.

### 4.4 Food assistance and “food is medicine” models

Food assistance can be integrated into cancer care through grocery vouchers, medically tailored meals, food pharmacies, produce prescriptions, meal delivery, partnerships with food banks, and community kitchens. For women undergoing treatment, food assistance should consider symptom patterns, protein requirements, food safety, cultural preferences, and household composition. For survivors with obesity or metabolic risk, support should emphasize dietary quality rather than calorie provision alone.

A “food is medicine” approach is particularly relevant in cancer care because nutritional barriers directly affect treatment tolerance, recovery, and survivorship health (12). Yet such programs require governance: eligibility criteria, sustainable financing, coordination with clinical teams, quality standards, and outcome evaluation. Philanthropic models can be useful but may be unstable; policy-level integration with insurance, public health, and social assistance systems is needed for sustainability.

### 4.5 Digital and hybrid community platforms

Digital tools can support community-governed nutrition care by enabling symptom tracking, dietary education, appointment reminders, referral coordination, and remote dietitian consultation. For women in high-density cities, digital platforms can link patients to nearby food resources, community services, and survivorship programs. For rural patients, tele-nutrition may reduce travel burden.

However, digital health can also widen disparities if older adults, low-income women, rural residents, migrants, or those with limited digital literacy are excluded. Hybrid models are preferable: digital tools should be combined with community health workers, telephone support, printed materials, and primary-care follow-up. Community governance should ensure that digital nutrition programs are accessible, multilingual, culturally appropriate, privacy-protective, and connected to real services rather than functioning as standalone apps. Evidence for digital nutrition support is promising, but implementation failures may occur when platforms are not reimbursed, not embedded in clinical workflows, or not usable by the women at greatest nutritional risk.

## 5 Policy pathways to reduce survival disparities

The first policy priority is to make nutrition screening a standard quality indicator in women's cancer care. Screening should include not only body weight but also food insecurity, dietary intake, treatment-related symptoms, and social barriers (34, 36). Institutions should report whether positive screens result in referral or assistance.

The second priority is to finance nutrition care. Dietitian services, medical nutrition therapy, food assistance, and community navigation are often underfunded. If these services are not reimbursed or supported by public programs, they will remain unavailable to the women most likely to need them. Payment models should recognize nutrition care as part of oncology treatment and survivorship, not as an optional wellness service.

The third priority is to build local service networks. Cancer centers should establish formal partnerships with primary-care clinics, public health agencies, community organizations, food banks, women's associations, and social care providers. These partnerships should define referral processes, feedback loops, data-sharing rules, and accountability measures.

The fourth priority is workforce development. Oncology clinicians, nurses, community health workers, and primary-care providers require training in nutrition risk recognition, food insecurity screening, culturally sensitive counseling, and referral. Dietitians should be integrated into multidisciplinary oncology and survivorship teams (35).

The fifth priority is equity monitoring. Community governance should track who receives nutrition services, who is missed, and whether outcomes differ by income, geography, race or ethnicity, age, insurance status, migrant status, and rurality. Without equity metrics, service expansion may benefit women who already have better access.

Implementation feasibility remains a major constraint. Financing may be contested because nutrition counseling, food assistance, and navigation are often distributed across medical, public health, and social-service budgets. Workforce shortages can limit dietitian access, especially in community and rural oncology settings. Governance conflicts may arise when hospitals, local governments, charities, and food providers have different accountability standards. These barriers should be anticipated rather than treated as secondary details.

**Figure 2:**
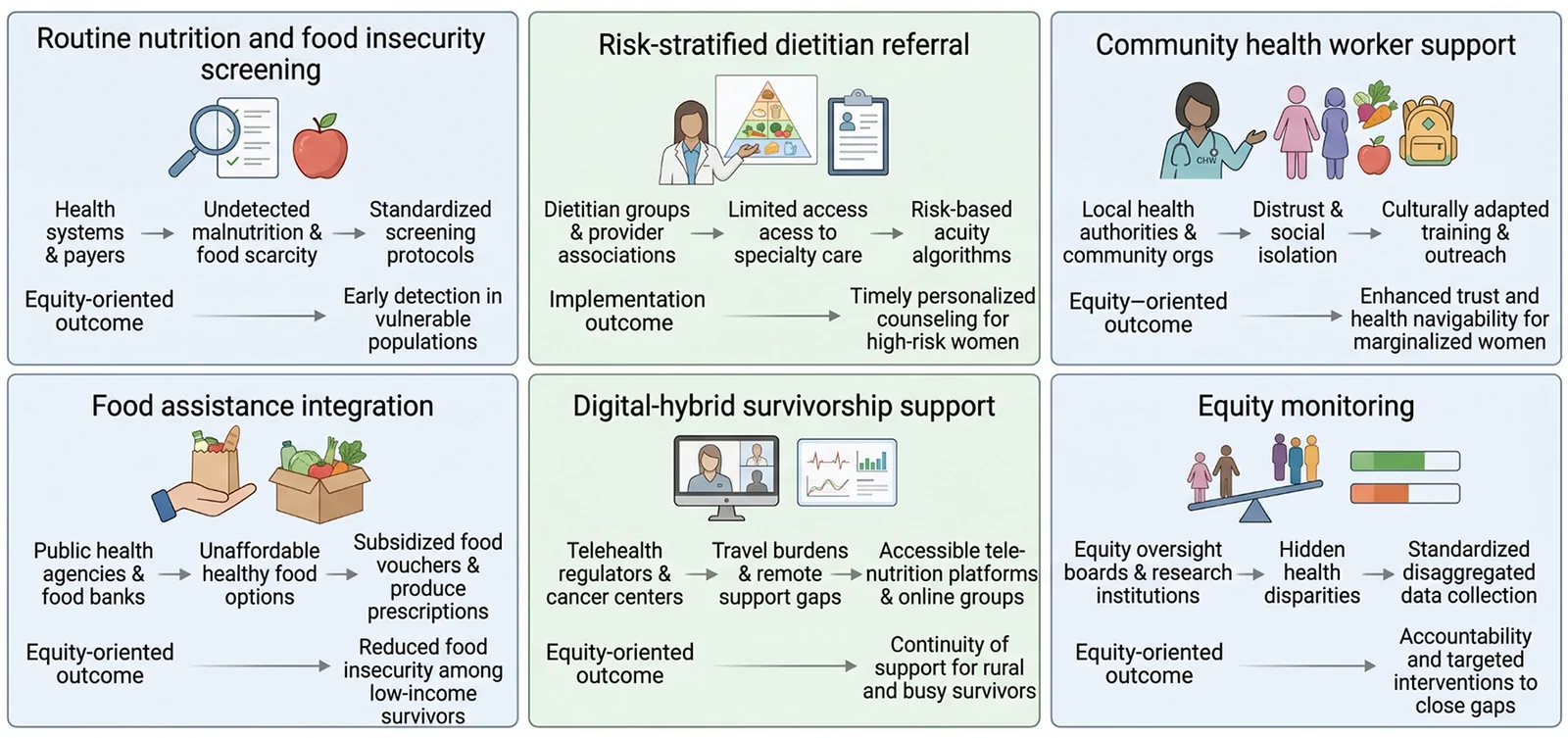
Policy pathways for community-governed nutrition-oriented cancer care for women. Six pathways are highlighted: routine nutrition and food insecurity screening, risk-stratified dietitian referral, community health worker support, food assistance integration, digital-hybrid survivorship support, and equity monitoring. Policy success is defined by timely and effective care for nutritionally vulnerable women.

A policy pathway for translating community governance into nutrition-oriented cancer care is presented in Figure 2.

## 6 Discussion: from community programs to accountable systems

Community governance reframes nutrition-oriented cancer care as an accountable system rather than a collection of disconnected programs. The central question is not whether nutrition advice exists, but whether women facing the highest nutritional and social risk can obtain effective support in time to influence treatment tolerance, recovery, and survivorship health. This is particularly important for women with breast, cervical, ovarian, and endometrial cancers, whose outcomes are shaped by both tumor biology and social determinants of health.

The evidence base remains incomplete and uneven. Individualized dietitian counseling and guideline-based malnutrition management are supported by stronger clinical nutrition rationale, whereas community food assistance, navigation, and digital-hybrid models remain more dependent on implementation evidence and context-specific adaptation. Some programs may improve food security, quality of life, or treatment completion without yet demonstrating reduced hospitalization or survival benefit. This does not negate their value, but it requires precise claims about what each intervention has shown and what remains uncertain.

Future research should use pragmatic and comparative designs to test which governance components are necessary, scalable, and cost-effective. Priority gaps include randomized or quasi-experimental evaluations of food assistance in oncology, community health worker models for nutrition follow-up, risk-stratified referral algorithms, digital access strategies for older and rural women, and equity dashboards that report who is reached and who remains underserved. Outcomes should include nutritional risk, body composition, dietary quality, treatment completion, symptom burden, hospitalization, quality of life, financial strain, and survival-related endpoints. Mechanistic anchoring would further strengthen the field by linking community-level interventions to metabolic, inflammatory, microbiome, or body-composition biomarkers where feasible.

## 7 Conclusion

Nutrition-oriented cancer care for women cannot be delivered effectively through clinic-based advice alone. Nutritional vulnerability is shaped by food insecurity, unequal food environments, fragmented care pathways, gendered caregiving roles, low nutrition literacy, and limited access to dietetic services. Community governance offers a practical framework for linking oncology, primary care, public health, social care, food systems, digital platforms, and patient organizations. Its contribution is not to replace integrated care, but to make local nutrition support accountable for screening, referral, food access, follow-up, and equity-sensitive outcomes. The goal is not merely to increase the volume of services, but to ensure that women at highest nutritional and social risk receive timely, culturally appropriate, and outcome-oriented support capable of reducing survivorship disparities.

## References

[1] Sung H Ferlay J Siegel RL Laversanne M Soerjomataram I Jemal A . Global Cancer Statistics 2020: GLOBOCAN estimates of incidence and mortality worldwide for 36 cancers in 185 countries. CA Cancer J Clin. (2021) 71:209–49. doi: 10.3322/caac.21660 33538338

[2] Rock CL Thomson CA Sullivan KR Howe CL Kushi LH Caan BJ . American Cancer Society nutrition and physical activity guideline for cancer survivors. CA Cancer J Clin. (2022) 72:230–62. doi: 10.3322/caac.21719 35294043

[3] World Cancer Research Fund/American Institute for Cancer Research. Diet, nutrition, physical activity and cancer: a global perspective. Continuous Update Project Expert Report. London: WCRF International (2018).

[4] World Cancer Research Fund International. Diet, Nutrition, Physical Activity and Body Weight for People Living with and Beyond Breast Cancer. London: WCRF International (2024).

[5] Arends J Bachmann P Baracos V Barthelemy N Bertz H Bozzetti F . ESPEN guidelines on nutrition in cancer patients. Clin Nutr. (2017) 36:11–48. doi: 10.1016/j.clnu.2016.07.015 27637832

[6] Muscaritoli M Arends J Bachmann P Baracos V Barthelemy N Bertz H . ESPEN practical guideline: clinical nutrition in cancer. Clin Nutr. (2021) 40:2898–913. doi: 10.1016/j.clnu.2021.02.005 33946039

[7] Cederholm T Jensen GL Correia MITD Gonzalez MC Fukushima R Higashiguchi T . GLIM criteria for the diagnosis of malnutrition: a consensus report. Clin Nutr. (2019) 38:1–9. doi: 10.1016/j.clnu.2019.02.033 30181091

[8] Fearon K Strasser F Anker SD Bosaeus I Bruera E Fainsinger RL . Definition and classification of cancer cachexia: an international consensus. Lancet Oncol. (2011) 12:489–95. doi: 10.1016/S1470-2045(10)70218-7 21296615

[9] Baracos VE Martin L Korc M Guttridge DC Fearon KCH. Cancer-associated cachexia. Nat Rev Dis Primers. (2018) 4:17105. doi: 10.1038/nrdp.2017.105 29345251

[10] Ligibel JA Bohlke K May AM Clinton SK Demark-Wahnefried W Gilchrist SC . Exercise, diet, and weight management during cancer treatment: ASCO guideline. J Clin Oncol. (2022) 40:2491–507. doi: 10.1200/JCO.22.00687 35576506

[11] Chan DSM Vieira AR Aune D Bandera EV Greenwood DC McTiernan A . Body mass index and survival in women with breast cancer—systematic literature review and meta-analysis. Ann Oncol. (2014) 25:1901–14. doi: 10.1093/annonc/mdu042 24769692 PMC4176449

[12] Raber M Jackson A Basen-Engquist K Bradley C Chambers S Gany FM . Food insecurity among people with cancer: nutritional needs as an essential component of care. J Natl Cancer Inst. (2022) 114:1577–83. doi: 10.1093/jnci/djac135 36130287 PMC9745434

[13] McDougall JA Anderson J Adler Jaffe S Guest DD Sussman AL Meisner ALW . Food insecurity and forgone medical care among cancer survivors. JCO Oncol Pract. (2020) 16:e922–32. doi: 10.1200/JOP.19.00736 32384017 PMC7489488

[14] Gundersen C Ziliak JP. Food insecurity and health outcomes. Health Aff. (2015) 34:1830–9. doi: 10.1377/hlthaff.2015.0645 26526240

[15] National Cancer Institute. Addressing the social needs of people with cancer. Cancer Currents Blog. Bethesda: National Cancer Institute (2024).

[16] World Health Organization. Framework on Integrated, People-Centred Health Services. Geneva: WHO (2016).

[17] National National Academies of Sciences Engineering and Medicine. Integrating Social Care Into the Delivery of Health Care: Moving Upstream to Improve the Nation's Health. Washington, DC: National Academies Press (2019). 31940159

[18] Alderwick H Gottlieb LM. Meanings and misunderstandings: a social determinant of health lexicon for health care systems. Milbank Q. (2019) 97:407–19. doi: 10.1111/1468-0009.12390 31069864 PMC6554506

[19] Wagner EH. Chronic disease management: what will it take to improve care for chronic illness? Eff Clin Pract. (1998) 1:2–4. 10345255

[20] Valentijn PP Schepman SM Opheij W Bruijnzeels MA. Understanding integrated care: a comprehensive conceptual framework based on the integrative functions of primary care. Int J Integr Care. (2013) 13:e010. doi: 10.5334/ijic.886 23687482 PMC3653278

[21] National Cancer Institute. Nutrition in Cancer Care (PDQ^®^)–Health Professional Version. Bethesda: National Cancer Institute (2024).

[22] Perry HB Hodgins S Crigler L LeBan K. Community health worker programmes after the 2013–2016 Ebola outbreak. Bull World Health Organ. (2016) 94:551–3. doi: 10.2471/BLT.15.164020 27429495 PMC4933142

[23] Patel MI Kapphahn K Dewland M Aguilar V Sanchez B Sisay E . Effect of a community health worker intervention on acute care use in advanced cancer: a randomized clinical trial. JAMA Oncol. (2022) 8:1139–48. doi: 10.1001/jamaoncol.2022.1997 35771552 PMC9247857

[24] Lewin S Munabi-Babigumira S Glenton C Daniels K Bosch-Capblanch X van Wyk BE . Lay health workers in primary and community health care. Cochrane Database Syst Rev. (2010) 2010:CD004015. doi: 10.1002/14651858.CD004015.pub3 20238326 PMC6485809

[25] Kangovi S Mitra N Grande D White ML McCollum S Sellman J . Patient-centered community health worker intervention to improve posthospital outcomes. JAMA Intern Med. (2014) 174:535–43. doi: 10.1001/jamainternmed.2013.14327 24515422

[26] Okasako-Schmucker DL Peng Y Cobb J Buchanan LR Xiong KZ Mercer SL . Community health workers to increase cancer screening: 3 Community Guide systematic reviews. Am J Prev Med. (2023) 64:579–94. doi: 10.1016/j.amepre.2022.10.016 36543699 PMC10033345

[27] Pérez-Escamilla R Shamah-Levy T Candel J. Food security governance in Latin America: principles and the way forward. Glob Food Sec. (2017) 14:68–72. doi: 10.1016/j.gfs.2017.07.001

[28] Ottery FD. Definition of standardized nutritional assessment and interventional pathways in oncology. Nutrition. (1996) 12:S15–9. doi: 10.1016/0899-9007(96)90011-8 8850213

[29] Kondrup J Rasmussen HH Hamberg O Stanga Z. Nutritional risk screening (NRS 2002): a new method based on an analysis of controlled clinical trials. Clin Nutr. (2003) 22:321–36. doi: 10.1016/S0261-5614(02)00214-5 12765673

[30] Ravasco P. Nutrition in cancer patients. J Clin Med. (2019) 8:1211. doi: 10.3390/jcm8081211 31416154 PMC6723589

[31] World Health Organization. Community Engagement: A Health Promotion Guide for Universal Health Coverage in the Hands of the People. Geneva: WHO (2020).

[32] National National Academies of Sciences Engineering and Medicine. Cancer Care for the Whole Patient: Meeting Psychosocial Health Needs. Washington, DC: National Academies Press (2008). 20669419

[33] Gany F Bari S Crist M Moran A Rastogi N Leng J. Food insecurity: limitations of emergency food resources for our patients. J Urban Health. (2013) 90:552–8. doi: 10.1007/s11524-012-9750-2 22829107 PMC3665978

[34] Jones AD Ngure FM Pelto G Young SL. What are we assessing when we measure food security? A compendium and review of current metrics. Adv Nutr. (2013) 4:481–505. doi: 10.3945/an.113.004119 24038241 PMC3771133

[35] Basen-Engquist K Alfano CM Maitin-Shepard M Thomson CA Schmitz K. Agenda for translating physical activity, nutrition, and weight management interventions for cancer survivors into clinical and community practice. Obesity. (2017) 25:S9–S22. doi: 10.1002/oby.22031 29086526 PMC5683412

[36] Bickel G Nord M Price C Hamilton W Cook J. Guide to Measuring Household Food Security. Alexandria, VA: US Department of Agriculture. (2000).

